# A review of the contribution of cowpea leaves to food and nutrition security in East Africa

**DOI:** 10.1002/fsn3.1337

**Published:** 2019-12-17

**Authors:** Joshua O. Owade, George Abong’, Michael Okoth, Agnes W. Mwang’ombe

**Affiliations:** ^1^ Department of Food Science, Nutrition and Technology University of Nairobi Nairobi Kenya; ^2^ Department of Plant Science and Crop Protection University of Nairobi Nairobi Kenya

**Keywords:** Acceptability, Cowpea Leaves, Nutritional Quality, Preservation

## Abstract

Cowpea leaf is among the African indigenous vegetables that have been recommended for possible alleviation of food and nutrition insecurity in sub‐Saharan Africa (SSA). The vegetable is rich in micronutrients including iron and vitamin A whose deficiencies are prevalent in SSA. Considering the limitation of seasonal availability, preservation techniques have been adopted to enhance availability with little success. This review aims at highlighting the contribution of cowpeas leaves to food and nutrition security as well as research gaps that must be addressed to promote the utilization of value‐added forms that would have extended effect of improving its production and consumption. It was found that preserved and fresh cowpea leaves were rich in beta‐carotene and iron in the ranges of 0.25–36.55 and 0.17–75.00 mg/100 g dry weight, respectively. The proportion of rural households incorporating the vegetable in its various forms in the region can be as high as 30%. With adequate utilization, the vegetable provided up to ≥ 75% and 25% of RDAs for vitamin A and iron, respectively, of children aged 4–8. However, the utilization of preserved forms faced a limitation for a deviation of up to 30% in their sensory scores and decreased nutrient content as compared to the fresh ones hugely hindered their market penetration. Utilization of novel processing techniques incorporating concept of hurdle technology can help address these quality losses. In conclusion, preservation of cowpea leaves should seek not only to enhance the shelf‐life, but also to enhance acceptability of the products with a view of increased utilization.

## Graphical abstract

The consumption of the fresh cowpea leaves was high. The intake of the preserved form of cowpea leaves such as the dehydrated forms was low. The preservation techniques have altered sensory and nutritional quality. Hurdle technology was found to have the most promising outcome due to the higher retention of nutrient and sensory qualities of the vegetables.

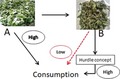

## 1 INTRODUCTION

Cowpeas (*Vigna unguculata* L. Walp) belong to the Family Fabaceae (Ibrahim, Satish, Ajay, & Karunakara, 2017; OECD, 2016). Production of cowpeas is both for its grains and vegetables. It is among the recognized African indigenous nutrient‐rich vegetables with the potential to promote food and nutrition security in sub‐Saharan Africa (SSA). The crop is originated from West and Central Africa, from where its cultivation and production spread to Latin America and South East Asia (Ano & Ubochi, 2008; Edeh & Igberi, 2012). The 2016 global estimates show that 12.3 million hectares of land are utilized in the production of cowpeas with Western and Eastern Africa leading in terms of production area at 10.5 and 0.9 million hectares, respectively (FAOSTAT, 2019).

Cowpea leaves have been exploited for food and feed. They are rich in micronutrients, nutraceuticals, and antioxidants. Some of the antioxidants that have been found in the leaves are alpha tocopherols, flavonoids, lycopene, and anticancer agents (Shetty, Magadum, & Managanvi, 2013). Cowpea vegetables contain important nutrients including vitamins and minerals that can improve the nutritional status of individuals and households with proper utilization (Okonya & Maass, 2014). The rich nutritional property of cowpea leaves makes them ideal for efforts aimed at reducing food and nutrition insecurity.

The burden of malnutrition in SSA is pronounced especially among the under five‐year‐old children and pregnant mothers (Stevens et al., 2015). Dietary diversification has been recommended as one of the effective strategies in combating vitamin A deficiency (VAD) and other micronutrient deficiencies in SSA. Cowpea leaves as one of the African indigenous vegetables have been incorporated into diets to improve food and nutrition security of the communities (Shiundu & Oniang'o, 2007). However, there is still limited utilization of the cowpea vegetables as it has a short shelf‐life thus limiting its availability.

Drying and other preservation methods have been recommended as possible ways of extending the shelf‐life and increasing availability of cowpea vegetables (Kiremire, Musinguzi, Kikafunda, & Lukwago, 2010). Over the ages, traditional African communities have subjected cowpea leaves to customized techniques for its preparation and storage. However, some of these processing techniques have deleterious effects on the nutritional composition and sensory quality of the cowpea leaves. Through research, modern techniques of preparation and preservation of cowpea leaves have been developed. The success in terms of increased utilization of cowpea leaves has still not been satisfactory. Questions have been raised with regard to the affordability and feasibility of some of these techniques considering that the production of cowpea leaves is mainly in the small scale. The current review focuses on the trend of utilization of cowpea leaves, the constraints in value addition and prospects to expand its utilization among communities. The review seeks to point out research gaps that can be exploited to improve the quality of preserved cowpea leaves and overcome the constraints that have greatly limited the utilization of cowpea leaves and its contribution to food and nutrition security.

## 2 METHODOLOGY

A comprehensive literature search was done in the Google Scholar database in order to capture relevant grey literature. A search syntax was (cowpea or Vigna AND unguiculata) “AND leaves AND (food OR nutrition) AND security AND production AND availability AND accessibility AND utilization AND stability)” without a restriction on the publication year. The first 300 articles from the Google Scholar as recommended by Haddaway, Collins, Coughlin, and Kirk (2015) were downloaded, and the abstracts were scrutinized for relevant literature. From these criteria, 86 articles were selected for the review. Six additional reports were extracted from the web pages by Food and Agriculture Organization and Government agencies including Horticultural Crops Directorate of Kenya.

## 3 RESULTS AND DISCUSSION

### 3.1 Production of cowpeas

The global estimates by Food and Agriculture Organization of the United Nations (FAO) on cowpea production only relate to the cowpea grains with Western Africa as the largest producer in the last half a decade as shown in Table 1. The estimates show that Western Africa accounted for 83.4% of the 6.99 million tonnes of global production in 2016. The crop is drought tolerant and warm weather crop that adapt well to drier areas of the tropics where other legumes cannot grow (Rashid, Hussain, Rahman, Khatun, & Sattar, 2016). The production of this crop in West Africa is both for domestic consumption and sale (Akpalu, Salaam, Oppong‐Sekyere, & Akpalu, 2014). The crop is mainly cultivated in mixed farming with cereals such as sorghum and millet due to its shade tolerance characteristics (Agbogidi, 2010). This has enabled cowpea vegetables and grains not only to aid in dietary diversification but also to serve as a security food in case of failure of the main crop. Cultivation of the crop can be done in soils that are poor as the crop has the ability to fix nitrogen for utilization in growth thereby encouraging its production by farmers in SSA (Edeh & Igberi, 2012; Horn, Ghebrehiwot, & Shimelis, 2016). In most places, the production is mainly in subsistence agriculture and on a small scale (Saidi, Itulya, Aguyoh, & Ngouajio, 2010).

**Table 1:** Global production of cowpea grains in tonnes for the period 2012–2016

Region	2012	2013	2014	2015	2016
Africa	8,054,899	8,030,197	5,357,312	5,552,211	6,739,689
Eastern Africa	510,357	474,479	490,131	471,779	465,687
Middle Africa	213,131	233,444	244,766	253,993	262,272
Northern Africa	39,000	79,000	101,100	54,148	172,162
Southern Africa	5,700	5,710	5,705	5,679	5,664
Western Africa	7,286,711	7,237,564	4,515,611	4,766,612	5,833,904
Americas	93,403	79,231	69,849	75,358	80,458
Asia	185,805	147,121	146,698	147,146	142,695
Europe	23,833	25,099	25,652	25,389	28,332
**World**	**8,357,941**	**8,281,648**	**5,599,511**	**5,800,105**	**6,991,174**

The production of cowpeas is spread across Asia, Europe, Africa, and America (Carvalho et al., 2017; De Souza, Farias, Lima, Ramos, & DeSousa, 2017). However, cowpea still remains a minor crop across Europe with most of the consumed vegetable being imported. Of the developed countries, only USA is a substantial producer and exporter of cowpeas (Directorate Plant Production, 2014). Asia has for a long period of time ranked second to Africa in terms of production of cowpeas (Nedumaran et al., 2015).

Cowpeas are mainly grown for subsistence in SSA with only a small proportion entering the international market (Directorate Plant Production, 2014). Young leaves and immature pods and seeds have been exploited as vegetables, whereas mature seeds have been consumed as pulses. However, harvesting of the leaves has been shown to affect the mean yield of the seeds. Kabululu, Ojiewo, Oluoch, and Maass (2014) reported a mean yield for cowpea leaves of 25 g/plant/two‐week harvest period accompanied by a 59% on farm reduction in grain yield for a local cowpea variety in Tanzania. Similar findings were also reported by Matikiti, Chikwambi, Nyakanda, and Mashingaidze (2009) where termination of leaf harvesting 7 weeks after crop emergence (WACE) resulted in 50.1%–70.4% reduction in grain yield. On the other hand, increasing the frequency of harvesting the leaves from 7‐day interval to 14‐day intervals was noted to increase the leaf yields by up to 100 percent (Saidi et al., 2010).

### 3.2 Cultivation of cowpeas in Kenya and East Africa

In East Africa, cultivated cowpeas are a source of vegetables and grains for human consumption. The major cowpea producing countries in East Africa include Kenya, Tanzania, South Sudan, and Uganda (USAID, 2010). The cowpeas that have been utilized across the region include both the landraces and improved varieties (Mamiro, 2011). A study done in Tanzania found that only 11% of the total cowpea leaves harvested ended up in the market for sale (Putter, Koesveld, & Visser, 2007). The average quantity of cowpea leaves sold per salesperson in the market per day in Tanzania was reported as 5.6 kg per day (Lotter, Marshall, Weller, & Mugisha, 2014). This quantity is low when compared to the amount sold per salesperson for other traditional vegetables such as amaranth (7.3 kg), nightshade (8.1 kg), cassava (6.1 kg), and ipomoea leaves (6.8 kg).

**Figure 1:**
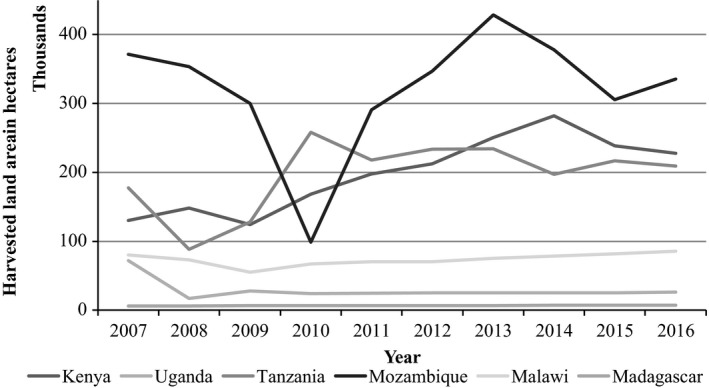
Trend of cultivation of cowpea in East and Central Africa. Adapted from (FAOSTAT, 2019)

**Figure 2:**
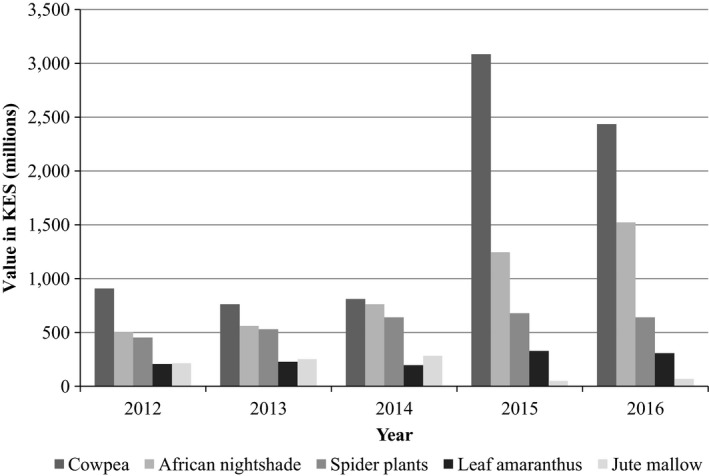
Value of some of the most utilized African indigenous vegetable in KES. Adapted from Horticultural Crops Directorate (2016) and (USAID and AFFA, 2014)

Kenya currently has the largest land area under cultivation of cowpea leaves in East Africa, standing at 227,809 hectares (FAOSTAT, 2019). The major cowpea production areas in Kenya include Bungoma, Kakamega, Kisumu, Siaya, Migori, Kitui, Makueni, Machakos, Kilifi, Kwale, and Tharaka Nithi Counties (Muniu, 2017). Kenya has steadily had an increasing trend in the cultivation of cowpea with the largest area under cultivation realized in 2014 in recent years (Figure 1). A report by Horticultural Crops Directorate in 2016 rated cowpea leaves as the most cultivated African indigenous leafy vegetable (AIV) in Kenya; ranking second in Eastern and Central Africa region (Figure 2); and contributing up to 43% of the total value of AIVs (Horticultural Crops Directorate, 2016). The cowpea varieties that have been cultivated in Kenya include landraces such as *Khaki*, *Macho*, *Kaima‐koko*, *Kutambaa*, *Mwandato*, *Nyekundu*, and *Nyeupe* and improved varieties such as KVU 419, Katumani 80, M‐66, *Kenya‐Kunde,* and KVU 27‐1 (Nderi & Kamau, 2018; Ndiso, Chemining, Olubayo, & Saha, 2016; Oyoo et al., 2017). Some of the cowpea varieties that have been grown in Tanzania and Uganda include the improved varieties such as *Ex‐Iseke*, KOL42 (UG‐CP‐9), *Dakawa*, IT93K204529, IT85F‐2841, MU‐93, and IT82D‐889 (Bisikwa et al., 2014; Kabululu, 2008). Other cowpea landraces spread across East Africa include *Cirikukwai and Ebelat.*

The production of cowpeas in East African region is mainly done in the arid and semi‐arid lands (ASALS) (Wambugu & Muthamia, 2009). In Kenya, Makueni County ranks the top in terms of cowpea leaves production area and quantity as shown in Table 2. The typical production area per person in Kenya ranges between 0.25 and 1 ha (Kiambi & Mugo, 2016). A study in Machakos and Tharaka Nithi Counties, known for their semi‐arid conditions, ranked the crop as the third and second most cultivated, respectively, in the two areas (An, Rm, & Lutta, 2016). The study also found that the farmers are willing to allocate up to 10% of their land for the production of cowpeas. The greatest constraint in the production of cowpea leaves currently being experienced in Kenya is that it is majorly limited to small‐scale farming (Ndungu et al., 2018).

**Table 2:** Performance of cowpeas in top producing counties in Kenya for the years 2013–2016

County	Year 2013		Year 2014		Year 2015		Year 2016	
	Area (Ha)	Volume (MT)	Area (Ha)	Volume (MT)	Area (Ha)	Volume (MT)	Area (Ha)	Volume (MT)
Makueni	Na	na	na	na	6,850	52,355	6,770	42,076
Kwale	217	878	1,122	6,982	4,337	18,845	4,130	13,612
Kitui	12,800	15,310	13,000	15,470	3,600	14,700	2,520	12,520
Machakos	1,593	5,441	2,668	4,814	3,629	8,280	3,945	9,642
Kilifi	Na	na	na	na	4,648	9,186	6,030	9,355
Taita Taveta	Na	na	na	na	549	4,037	580	4,133
Homa Bay	Na	na	na	na	639	2,442	927	3,613
Siaya	407	2,304	1,039	3,029	2,800	7,925	1,291	3,205

Note

na—data not available. Adapted USAID and AFFA (2014) and Horticultural Crops Directorate (2016).

In areas where it is grown in East Africa, the availability of cowpea leaves is along the cropping seasons (Okonya & Maass, 2014). The crop is mostly intercropped with cereals such as sorghum and maize.

### 3.3 Utilization of cowpea leaves

The underexploitation and lack of proper utilization of cowpea leaves and other traditional leafy vegetables has been a major undoing in the promotion of food security in sub‐Saharan Africa (SSA). A study by Madodé et al. (2011) cited the limited shelf‐life of cowpea leaves of just 24 hr at ambient temperatures as a major bottleneck to its utilization. For promotion of utilization of cowpea vegetables, extension of the shelf‐life, optimization of production and processing techniques, and reduction in postharvest losses have been recommended as possible strategies (Kirakou, Margaret, Ambuko, & Owino, 2017).

Harvesting of cowpea leaves usually begins as early as two weeks after emergence (WEA) and continues until flowering (Saidi et al., 2010). There is massive utilization of cowpea leaves during glut but less utilization during acute shortages in times of drought (Okello, Hutchinson, Mwang’ombe, Ambuko, Olubayo, 2015). This has been due to the limited shelf‐life of the cowpea vegetables. The high moisture content of cowpea leaves renders them highly perishable, while their seasonality in supply has resulted in limited utilization all year round (Njoroge, Matofari, Mulwa, & Anyango, 2016). Moreover, most consumers utilize the vegetable in its fresh form and make less use of the value‐added products.

Cowpea leaves have also been consumed as boiled, blanched, dried, or fermented vegetables (Kasangi, Shitandi, Shalo, & Mbugua, 2010; Kirakou et al., 2017; Muchoki, Imungi, & Lamuka, 2010). Among West African communities, these vegetables are consumed as accompaniment with cereals or as vegetable sauces with other foods such meat and fish (Madodé et al., 2011). In Kenya, cowpea leaves are consumed as potherbs just as other AIVs (Kanali et al., 2017). The utilization of cowpea leaves in its different forms in the rural areas in Tanzania is to the tune of 30% of the households (Ochieng et al., 2016). In Asia, the leaves are boiled then sun‐dried and stored for later use (Zia‐Ul‐Haq, Ahmad, Amarowicz, & Feo, 2013). This aims at beating the constraints of seasonality in terms of availability of cowpea leaves. The utilization of cowpea leaf concentrates, which are known to be rich in micronutrients, has been one of the modern techniques of its utilization (Jethwani, Dutta, & Singh, 2015). Other value‐added products of cowpea leaves that have been used in high‐end markets such as supermarkets include the vacuum‐packed, solar‐dried, powder, canned, and frozen forms (Jethwani et al., 2015; Okello et al., 2015; Onyeoziri, Kinnear, & Kock, 2018). However, the incorporation of cowpea leaves into other renowned and widely acceptable products as it has been done with other indigenous and underutilized crops, for example, orange‐fleshed sweet potato roots, remains less explored (Owade et al., 2018b).

Cowpea leaves have also been utilized as fodder for livestock (Mahama, 2012). They are recommended as protein‐rich animal feeds to be incorporated in feeds during formulation (Martens, Tiemann, Bindelle, Peters, & Lascano, 2012). The utilization of cowpea leaves as livestock fodder is not a common practice in SSA as it is mainly exploited for food.

### 3.4 Postharvest losses of cowpea leafy vegetables

The AIVs are usually in scarce supply during dry seasons but plenty during rainy seasons that occasion high postharvest losses (Seidu et al., 2012). Postharvest losses for cowpea leaves and other AIVs in East Africa have been estimated to be as high as 30%–40% of the production quantity with some countries recording even higher figures (Babatola, Ojo, & Lawal, 2008). Gogo, Opiyo, Ulrichs, and Huyskens‐Keil (2018) reported postharvest losses exceeding half of the production quantity of cowpea leaves and other AIVs among some of the farmers in Kenya. Postharvest losses in these vegetables have largely been attributed to handling, production practices, distribution, and marketing dynamics (Ndirangu et al., 2017). The quality of cowpea leaves deteriorates with storage. Storage conditions based on their temperature affect the physicochemical attributes of cowpea leaves in terms of antioxidant properties, nutritional quality, color, and texture (Kirigia, Winkelmann, Kasili, & Mibus, 2018; Natabirwa, Mukiibi, Zziwa, & Kabirizi, 2016). Value addition of cowpea leaves through adoption of optimal preservation techniques is seen as a possible way of improving the shelf‐life while retaining the nutritional and sensory quality (Zheng et al., 2009).

Poor postharvest handling practices aggravate the losses, which may rise up to 70% (FAO & World Bank, 2010). Poor transport and distribution network are some of the underlying economic factors that may increase these losses (Shiundu & Oniang'o, 2007). Low temperature storage (4°C) improves retention of the phytochemicals in the fresh cowpea leaves, thus enhanced keeping quality compared with those stored at room temperature (50%–55% relative humidity) in which the phytochemicals strongly declined at 4 days (Kirigia et al., 2018). Most of the farmers of cowpea leaves still rely on traditional techniques for handling the vegetables along the value chain resulting in greater losses (Gogo et al., 2018). The smallholder farmers lack necessary facilities such as low temperature storage equipment and reliable transportation means for the postharvest handling of cowpea leaves, and thus, significant proportion of their produce lose the saleable value (Onyango & Imungi, 2007). These produce attract less returns in the market or are rejected at the high‐end markets such as supermarkets.

### 3.5 Nutrient contribution of cowpea leaves to human diet

Dietary diversification has been employed over time as a strategy in improving nutrition status of the population. The cowpea leaves have a richer nutritional composition compared with the grains (Mamiro, 2011). The utilization of cowpea leaves for food has mainly been done in various dishes, soups, and sauces (Imungi & Potter, 1983). Cowpea leaves even in their preserved forms are known to be rich in nutrients that are essential for life (Table 3). Cowpea leaves have also been recommended as possible sources of leaf protein to enrich the diet (Ghaly & Alkoaik, 2010). Cowpea leaves are known to be rich in proteins, vitamins such as provitamin A, folate, thiamin, riboflavin, and vitamin C, and minerals, such as calcium, phosphorus, and iron (Kirakou, 2014; Xiong et al., 2016). Okonya and Maass (2014) reported the crude protein and iron contents of fresh cowpea leaves on a dry mass basis to be as high as 33 g/100g and 379 µg/g, respectively. A study by Van Jaarsveld et al. (2014) reported that a 90g portion of cowpea leaves could meet ≥ 75% and 25%–50% RDAs for vitamin A and iron, respectively, for children 4–8 years. However, it is worth noting that the nutrient composition of cowpea leaves varies depending on the cultivar (Carvalho et al., 2012), and thus, cultivar selection should be carefully done.

**Table 3:** Nutritional composition of cowpea vegetables (mg/100g dry weight)

Nutrient	Cowpea leaves (per 100 g dry matter)			
	Raw fresh ^a, b, d, e, f, k^	Dried ^d, h^ (Solar‐ and sun‐dried)	Blanched ^b, h, i, j^	Fermented ^g, i^
Moisture (g)	85–90	7.04–7.35	12.0–15.02	6.31–7.29
Crude Protein (g)	28–42	29.09–39.24	4.0–31.86	28.07–29.40
Crude lipid (g)	9.00–10.26	1.31–2.28	4.33–12.91	1.68–1.92
Crude ash (g)	4.80–13.58	10.84–14.80	7.5–11.87	10.6–11.0
Crude fiber (g)	10.09–25.51	14.26–29.31	12.53–14.35	17.10–29.48
Energy value (kCal)	325.36–390.26	219.8–290.51	246.27–384.43	214–226.9
Micronutrients				
Beta‐carotene (mg)	32.74–36.55	0.25–24.76	19.21–20.35	0.8–30
Vitamin C (mg)	70–203	1.39–137.9	40.1–42.8	45
Iron (mg)	66–75	0.58–7.50	0.56–0.57	0.17–0.23
Calcium (mg)	17.1–39.87	1.40–25.1	24.3–24.6	1.27–1.28
Zinc (mg)	5.22–12.91	1.66–144.5	0.14–7.9	0.05–0.07

Note

Adapted from ^a^Nekesa (2016), ^b^Aathira et al. (2017), ^c^Ahenkora et al. (1998), ^d^Chikwendu et al. (2014), ^e^Belane and Dakora (2012), ^f^Kirakou (2014), ^g^Muchoki (2007), ^h^Kirakou et al. (2017), ^i^Kasangi et al. (2010), ^j^Oula et al. (2015), and ^k^Imungi and Potter (1983).

### 3.6 Antinutritional factors in cowpea leaves

Cowpea leaves have antinutritional factors such as oxalates, phytates, and nitrates which are known to have negative impact on the nutrient intake of individuals (Muchoki et al., 2010; Oulai, Zoue, & Niamke, 2015). Optimal processing and preservation techniques should seek to reduce or minimize the accumulation of these antinutritional factors as a way of ameliorating the nutritional quality. Some of the processing techniques that have been used successfully in reducing antinutrients in foods include fermentation, soaking, germination, debranning, and autoclaving (Ertop & Bektaş 2018). Removal or reduction in the antinutrients serves to improve the nutritional quality of the food by increasing the bioavailability of nutrients such as protein, calcium, iron, and zinc. Muchoki (2007) reported a reduction of 38.4% and 8.3%, respectively, in the nitrate and oxalate contents of dried cowpea leaves that were subjected to fermentation. Another study by Chikwendu, Igbatim, and Obizoba (2014) similarly reported a reduction of 33.3%, 73.9%, 85.9%, and 70.7% in the tannin, saponins, flavonoid, and polyphenols contents, respectively, for cowpea leaves that were parboiled, sun‐dried, and drained. The practice in the latter study may not be encouraged as it would also result in greater losses in the important nutrients especially the micronutrients. Any techniques aimed at reducing these antinutritional factors must have minimal deleterious effects on essential nutrients.

### 3.7 Traditional processing and preservation of cowpea leaves among East African Communities

There are different customized recipes among the communities growing the cowpea vegetables. In Kenya, cowpea leaves can be consumed stewed as vegetables or even as mixed dishes with other vegetables such as jute mellow (FAO & GoK, 2018). Cowpea leaves are a common delicacy among Kenyan communities with the Mijikenda consuming it boiled mixed with coconut milk (Okello et al., 2015). Other preparation techniques that cowpea leaves have been subjected to include boiling with lye (traditional salt) and milk and frying (Akello, 2014). The boiled vegetables are at times subjected to fermentation for about 48 hr before its consumption in certain communities or even sun‐dried and stored for later use. The per capita consumption of cowpea leaves in the producing areas in Tanzania was reported to be 41–200 g (Mamiro, 2011). The vegetables are consumed in varied forms including the preserved ones.

### 3.8 Fermentation

Fermentation of cowpeas has been in practice among traditional African communities for a long time. The overall aim of the fermentation process has been to improve on the shelf‐life of the cowpea leaves, while at the same time the nutritional quality has also been improved. Muchoki et al. (2010) reported a reduction of 71.9% in the nitrate content of cowpea vegetables subjected to fermentation. However, the fermentation techniques employed in these traditional practices are spontaneous and the product quality in terms of the nutritional and sensory is still varied and not optimal. Attempt has been made to try and standardize the fermentation processes of these vegetables. Kasangi et al. (2010) used fermentable sugars at the rate of 1%–3% to enhance the cowpea leaves fermentation process and achieved a crude fiber and ash contents of 16.29%–17.61% and 22.37%–22.61%, respectively. These values were higher than those he reported for cowpea leaves that were solar‐dried (12.76% and 13.08% crude fiber and ash contents, respectively) or blanched solar‐dried (11.76% and 9.49% crude fiber and ash contents, respectively). However, the study fell short of establishing the effect of such treatment on the sensory appeal and color changes of the vegetables. Muchoki et al. (2010) while using the one‐factor method had established the salt and sugar concentrations each at 3% as the optimal for cowpea leaves fermentation. However, this has been shown to ignore the influence of interactions of the factors under investigation on the response. Further work should consider incorporating the influence of the interactions of the factors on the product quality. It is also recommended that in fermentation of cowpea vegetables and fermentable sugar such as glucose or fructose should be added at the level of 2%–3%, followed by a starter culture; this gives a better product in terms of its quality compared with the cowpea leaves subjected to spontaneous fermentation (Wafula et al., 2016).

### 3.9 Sun drying

Utilization of sun drying as a technique of food preservation was reported by Nnadi, Chikaire, and Ezudike (2013) as the most practiced technique by people, 94% of households, in traditional communities in Nigeria. The dried vegetables have been utilized in Uganda to overcome the shortage of cowpea leaves during drought (Aleni, 2017). Sun‐dried leaves are at times first steamed before being dried (Directorate Plant Production, 2014). The method is known to have concentration effect on nutrients thus increases the nutrient density (Chikwendu et al., 2014). However, the limitation of this technique has been that it results in decreased micronutrient contents. Ndawula, Kabasa, and Byaruhanga (2004) reported a nutrient loss of 58% and 84% for β‐carotene and vitamin C, respectively, during open drying. In another study, Chikwendu et al. (2014) reported a decrease in the iron, zinc, calcium, iodine, and phosphorus contents by 90.3%, 87.1%, 96.5%, 73.8%, and 64.6%, respectively, for cowpea leaves that had been boiled and sun‐dried. Drying under a shade is recommended to minimize the deleterious effects on micronutrient content (Directorate Plant Production, 2014).

Sun drying is one of the preservation techniques that have been recommended to improve the availability of cowpea vegetables to aid in the promotion of food and nutrition security in SSA. Consumption of sundried cowpea leaves together with other traditional indigenous vegetables was shown to improve the mean serum retinol content by 25.9% in a 13‐week feeding trial study (Nawiri, Nyambaka, & Murungi, 2013). Thus, the preserved vegetables are recommended for amelioration of nutritional status of vulnerable populations.

### 3.10 Modernized cowpea leaves processing techniques

Processing of cowpea vegetables has been used to improve the nutritional and keeping qualities of the vegetables using modernized technologies with the aim of increasing their utilization. Some of the processing techniques that have been utilized include solar drying, freezing, freeze‐drying, blanching, and vacuum packaging (Okello et al., 2015). The degree of deterioration in the nutritional and sensory quality of these preserved products vary depending on the cooking methods and preservation techniques in use (Okonya & Maass, 2014). Kirakou et al. (2017) reported low retention of beta‐carotene and ascorbic acid at 52.78% and 20.24%, respectively, for cowpea vegetables that were blanched in salty water for 2 min at 94°C followed by solar dying. Conventional cooking of cowpea vegetables that entailed boiling for 10 min resulted into total loss of vitamin C (Rashid et al., 2016). Thus, the processing technique used should be carefully selected with the aim of maximum retention of the nutritional and sensory quality.

### 3.11 Blanching

Blanching has been used to preserve cowpea vegetables and extend their utilization. Variation in the blanching temperature–time combination has an effect on the nutritional composition and microbial quality of the vegetables (Njoroge et al., 2016). Oulai et al. (2015) reported β‐carotene and vitamin C losses of up to 55.5% and 61.1%, respectively, for cowpea vegetables blanched in a pressure cooker (121°C) for 15 min. Increasing the blanching time resulted in more nutrient losses, however, with a positive impact of reduction in antinutritional components. Aathira, Sudarsa, and Siddhuraju (2017) also reported a similar trend in the loss of protein in cowpea leaves blanched at 100°C for 20 min (35.4%) compared with those blanched at 100°C for 15 min (31.3%). Even so, blanching also has the positive impact of attenuating the deleterious effects of drying on antioxidant activity such as 2,2‐diphenyl‐2‐picryl hydrazyl (DPPH) scavenging activity; thus, blanched vegetables are highly recommended as alternative sources of antioxidants (Nobosse, Fombang, & Mbofung, 2017).

The other non‐nutritional benefit of blanching is that it improves the rehydrability, reduces microbial load, and increases the ease of packaging as it shrinks and softens the vegetables (Njoroge et al., 2016).

### 3.12 Solar drying

Solar drying has also been used in the preservation of cowpea leaves as a modern technique. The greatest concern with this preservation technique still remains the retention of micronutrients such as ascorbic acid and β‐carotene as it exposes these nutrients to oxidation in the presence of oxygen. Visqueen‐covered solar‐dried cowpea vegetables showed great losses for β‐carotene and vitamin C at 34% and 71%, respectively (Ndawula et al., 2004). Blanching of the vegetables before solar drying improved the retention of β‐carotene and vitamin C by 15% and 7.5%, respectively. This gives greater credence to the use of hurdle technology in the preservation of cowpea leaves.

### 3.13 The concept of hurdle technology

A combination of at least two preservation techniques has been reported to produce the best results in terms of nutritional and keeping quality. Njoroge et al. (2016) reported that blanching at 80°C for 10 min and hot‐air drying of cowpea vegetables had a retention of 53.7% and 53.8% for vitamin C and β‐carotene, respectively, whereas blanching at 80°C for 10 min and solar drying recorded a retention of 58.2% and 49.2% for vitamin C and β‐carotene, respectively. These values were higher when compared with that of conventional method of boiling at 100°C for 30 min. In the traditional preparation of cowpea leaves, blanching was not included in the preparatory processes prior to sun drying, and lower retention of β‐carotene was noted (Mulokozi & Svanberg, 2003). Without blanching, sundried vegetables are exposed to continued enzyme activity that would easily expose the carotenoids to oxidation reactions.

Utilization of a combination of different storage technologies and appropriate storage conditions can also improve the micronutrient retention. Anyango (2015) reported the least losses of 13.6%–13.8% in β‐carotene content of sun‐dried cowpea leaf vegetables under inert conditions as compared to losses of 56.3%–57.3% and 29.6%–33.3% under normal conditions and modified atmospheric packages, respectively. However, this too has had a fair share of challenges in achieving faultless results as storage conditions affected acceptability. A study by Natabirwa et al. (2016) that evaluated the acceptability of cowpea leaves that were blanched and then either sun‐dried or solar‐dried reported that the preserved products had lower acceptability scores (5.6–6.5) than the fresh cowpea vegetables (8.0) on a 9‐point hedonic scale. It is therefore important that these studies must also establish the impact of these technologies on the acceptability of the product.

### 3.14 Consumer acceptance of value‐added cowpea products

The greatest incentive for promotion of value addition practices is successful adoption of the products (Owade, Abong, Okoth 2018a). Increased utilization of cowpea leaves can only be achieved when the preserved cowpea leaves are marketable. A study by Okello et al. (2015) reported that Kenyan consumers were willing to pay as high as KES: 5 for both the sun‐dried and frozen cowpea leaves. The same study notes that some consumers attach no great significance on value addition practices and would not be willing to pay extra for such. The current low consumption can be attributed to wrong consumer perception about the cowpea leaves. Lekunze (2014) in his market analysis study of cowpea leaves reported that households who were regular consumers of the vegetables would more likely substitute these vegetables with other vegetables in the likely event that their income levels increased.

Another study that evaluated the acceptability of different cowpea leaves established that nutritional composition such as ascorbic acid, moisture and phosphorus content, and the leaf size was correlated with the acceptability of cowpea leaves (Ahenkora, Adu Dapaah, & Agyemang, 1998). Dehydration of cowpea leaves as a way of preservation results in alteration of the nutritional and thus the sensory quality too. Descriptive sensory analysis of dehydrated cowpea leaves revealed that the solar‐dried leaves had similar appearance and texture to the fresh cowpea leaves, whereas greater alterations existed in the sundried cowpea leaves (Nyambaka & Ryley, 2004). The greatest gap that has to be filled is to have the consumers adopt value‐added cowpea leaves.

### 3.15 Constraints of value addition practices for cowpea vegetables

The primary bottleneck for the utilization and value addition practices for cowpea leaves stems from the production practices. The production practices in the SSA for cowpea leaves are below the optimal levels, and thus, low production quantities have been realized (Oyewale & Bamaiyi, 2013). Some of agricultural practices also impoverish the soil thus affecting the nutrient content of cowpea leaves produced from these soils. All the above highlighted factors thereby result into lower economic returns that greatly discourage the production of cowpea leaves (Mucheru‐Muna et al., 2010).

The marketing system for cowpea leaves and other AIVs is mainly through the informal sectors where quality parameters are less observed (Onyango & Imungi, 2007). The seasonal nature of the supply of cowpea leaves makes its handling in the less organized informal market difficult resulting to major losses in form of spoilage (Omulo, 2016). These farmers rely on traditional preservation techniques such as storage under a shade and sprinkling of water on vegetables in stores which has its limitations as the keeping quality is only extended by a few days (Kirigia et al., 2018). The modernized cold storage facilities such as cold rooms are unaffordable to the smallholder farmers (Onyango & Imungi, 2007). Lack of time, inadequate knowledge, and additional costs have resulted into less practice of value addition among most handlers in the value chains (Kirui, Kisang, & Kiptum, 2017). A study in Malawi and Mozambique reported that the weak value chain linkages and less value addition practices for traditional vegetables including cowpea leaves as a major bottleneck in their marketing (Chagomoka, Afari‐Sefab, & Pitoroc, 2014). Another study in South Africa found that these farmers lacked technical knowledge and ability of value addition that would be important in improving their revenues (Senyolo, Wale, & Ortmann, 2018).

### 3.16 Future prospects

Future prospects of vegetables preservation are looking into techniques that retain most of the nutritional and sensory quality, minimize microbial contamination, and improve the shelf‐life of the vegetables. Zheng et al. (2009) in his study of vegetable dehydration found that a combination of centrifugation after washing and microwave drying with proper packaging of the material reduced dehydration time and improved nutritional and sensory quality. Minimizing drying time is recommended for maximum retention of physicochemical and sensory quality of the vegetables (Chege, Kuria, Kimiywe, & Nyambaka, 2014). Incorporation of centrifugation in the production of dehydrated vegetables can help reduce the drying time and thus improve the nutrient retention in dried vegetables. Value addition of the underutilized crops has the extended effect of increasing commercialization of the crop, providing an incentive for increased production.

Other vegetables have also been preserved through fermentation by dry salting techniques that largely retained the physical and chemical attributes (Vatansever, Vegi, Garden‐Robinson, & Hall, 2017). Such techniques can be extended to the preservation of cowpea leaves. However, such studies must establish optimal conditions for retention of nutrient and sensory quality of the products. Such a study would also need to establish the diffusion coefficient of salt into the vegetables during preservation as it has an influence on the physical attributes of the vegetables (Kusnadi & Sastry, 2012). The role of packaging in enhancing the keeping quality while maximizing the retention of nutritional and sensory quality of cowpea leaves still remains largely unexplored. Khatoniar and Barooah (2018) reported a higher efficiency in the preservation of dehydrated vegetables packaged in higher density polyethylene (HDPE) pouches, moisture penetration as low as 4.2% in storage period of six months, as compared to polypropylene pouches and plastic bottles. Polymeric films and antimicrobial packaging have been exploited as preservation techniques for other foods (Scetar, Kurek, & Galic, 2010), but are yet to be utilized in the preservation of cowpeas.

### 3.17 Gaps in knowledge

In spite of the many value‐added products of cowpea leaves in the market, the uptake is still very low. The practices are also very limited with a large proportion of the cowpea leaves still sold as fresh produce. In some instances, the less optimal traditional preservation techniques are preferred due to affordability. The focus of research should be to promote affordable techniques of preservation of cowpea leaves that are acceptable among the value chain actors that in turn will promote their utilization.

The quality in terms of nutritional and sensory quality of the value‐added cowpea leaves greatly deviates from those of fresh produce pointing to less optimal practices. There is need for research on optimization of some of these traditional techniques such as fermentation to develop highly acceptable products whose uptake in the market would be almost as much as the fresh cowpea leaves.

## 4 CONCLUSION

Cowpea is a popular crop in the SSA that is rich in nutrients and phytochemicals but is yet to be fully harnessed. Both the leaves and grain of cowpeas are exploited for food. The leaves are of high nutritional value and would be of great importance in strategies aimed at addressing food and nutrition security. However, the concerns of shelf stability of the fresh product and the nutritional and sensory quality of the processed product greatly limit the utilization of this vegetable. Some of the preserved products have poor sensory and nutritional quality as compared to the fresh leaves. Different preservation techniques are utilized to preserve cowpea leaves. The efficiency of these techniques in achieving different quality requirements has great variations. The use of hurdle technology provides better results as compared to utilization of preservation techniques separately. Some of these techniques are yet to be evaluated in the preservation of cowpea leaves.

Acceptability of a product hugely determines its uptake in the market and thus influences its successful adoption. The major focus in the preservation of these vegetables should not just be on the nutritional quality but must also consider the sensory appeal of the product. The preservation should seek to improve the sensory quality of the preserved cowpea leaves that largely influences its consumer acceptability.

## CONFLICT OF INTEREST

The authors declare that they do not have any conflict of interest.

## ETHICAL APPROVAL

The study did not involve any animal or human testing.

## INFORMED CONSENT

The study did not involve human subjects.
